# Treatment modification in HIV-Infected individuals starting antiretroviral therapy between 2011 and 2014

**DOI:** 10.7448/IAS.17.4.19768

**Published:** 2014-11-02

**Authors:** Michaela Rappold, Armin Rieger, Andrea Steuer, Maria Geit, Mario Sarcletti, Bernhard Haas, Ninon Taylor, Manfred Kanatschnig, Gisela Leierer, Bruno Ledergerber, Robert Zangerle

**Affiliations:** 1 Department of Dermatology and Venereology, Innsbruck Medical University, Innsbruck, Austria; 2 Department of Dermatology and Venereology, Medical University of Vienna, Vienna, Austria; 3 Department of Pulmonary Medicine, Otto Wagner Hospital, Vienna, Vienna, Austria; 4 Department of Dermatology and Venereology, Allgemeines Krankenhaus Linz, Linz, Austria; 5 Department of Infectious Diseases, Landeskrankenhaus Graz West, Graz, Austria; 6 Department of Internal Medicine III, Paracelsus Medical University Salzburg, Salzburg, Austria; 7 Department of Internal Medicine, Landeskrankenhaus Klagenfurt, Klagenfurt, Austria; 8 Division of Infectious Diseases, University Hospital Zurich, Zurich, Switzerland

## Abstract

**Introduction:**

While antiretroviral therapy (ART) has increased the survival of HIV patients and turned HIV infection into a chronic condition, treatment modifications and poor adherence might limit this therapeutic success.

**Methods:**

Patients from the Austrian HIV Cohort Study, who started their first ART after Rilpivirine became available in February 2011, were analyzed for factors associated with treatment modification which could be either a change of drugs or a stop of the regimen. A drug was considered as stopped when the regimen was interrupted for more than eight days. Drugs of particular interest were Darunavir (DRV), Atazanavir (ATV), Raltegravir (RAL), Rilpivirine (RPV) and Efavirenz (EFV). RPV and EFV were analyzed only when taken as single tablet regimen. Other drugs were summarized as “other.” Proportional hazards regression methods were used to identify predictors of discontinuation and Kaplan–Meier estimates were used to calculate probabilities of discontinuation. Patients who died were censored at the date of death.

**Results:**

965 patients started ART, 282 with DRV, 161 with ATV, 96 with RAL, 108 with RPV and 118 with EFV. Median time for taking initial ART is 11.6 months. 322 (33.4%) patients modified their initial ART. The overall probability of modification at one year was 28.7%, at two years 40.0% and at three years 49.8%. In a multivariable proportional hazards regression analysis, AIDS diagnosis at baseline and injecting drug use (IDU) of men compared with men who have sex with men (MSM) have a higher risk of switch/stop. Compared with DRV, RPV showed a much lower and ATV and particularly “other” a higher risk for discontinuation (Table 1).

Availability of more effective/convenient treatment (28.9%) was the main reason for discontinuation, especially in the group “other” (43.5%), RAL (34.6%) and DRV (31.6%). Non-specified patient or physician wish to modify therapy was revealed in 17.4% and 9.3% respectively. EFV was modified in 52.8% due to central nervous system toxicity and ATV in 27.8% for gastrointestinal toxicity including hyperbilirubinemia.

**Conclusion:**

Rates of modification and interruption were still high in recent years, particularly in the first year of ART. The decreased rate of modification found in patients treated with Rilpivirine may be attributed to selection of patients according to guidelines.

**Table 1:** Uni- and multivariable Cox regression: association between different baseline characteristics and modifications of the initial ART regimen

	Univariable Cox Regression		Multivariable Cox Regression	
	
	Hazard ratio	95% CI	Hazard ratio	95% CI
Age				
<30 years	0.98	0.68–1.42	0.98	0.66–1.47
30–50 years	0.89	0.63–1.26	0.89	0.62–1.27
≥50 years	1	Reference	1	Reference
HIV transmission category				
Male injecting drug user	1.62	1.14–2.29	1.45	1.02–2.06
Female injecting drug user	0.91	0.46–1.78	0.82	0.41–1.62
Male heterosexual	1.01	0.75–1.36	0.89	0.65–1.21
Female heterosexual	1.28	0.94–1.73	0.98	0.72–1.34
Other	0.78	0.41–1.48	0.54	0.28–1.04
Men who have sex with men	1	Reference	1	Reference
Drugs/Regimen				
RPV	0.15	0.06–0.42	0.16	0.06–0.45
EFV	1.04	0.70–1.54	1.03	0.69–1.54
ATV	1.75	1.27–2.42	1.68	1.21–2.32
RAL	1.20	0.77–1.88	1.09	0.69–1.72
Other	2.98	2.22–4.01	3.10	2.30–4.18
DRV	1	Reference	1	Reference
AIDS at baseline				
Yes	1.73	1.24–2.40	1.68	1.19–2.37
No	1	Reference	1	Reference

